# Supplementary material for: Similar temperature scale for valence changes in Kondo lattices with different Kondo temperatures
Source: Nat Commun. 2018 May 22;9:2011. doi: 10.1038/s41467-018-04438-8 (PMC5964219; doi:10.1038/s41467-018-04438-8)
Supplement: Supplementary file 1 — Supplementary Information [file 41467_2018_4438_MOESM1_ESM.pdf]

Similar temperature scale for valence changes in Kondo lattices with different Kondo temperatures

-Supplementary Information-

K. Kummer[1,*], C. Geibel[2], C. Krellner[2, 3], G. Zwicknagl[4], C. Laubschat[5], N. B. Brookes[1], and D. V. Vyalikh[6, 7, 8]

[1]European Synchrotron Radiation Facility, 71 Avenue des Martyrs, CS40220, F-38043 Grenoble Cedex 9, France
[2]Max Planck Institute for Chemical Physics of Solids, Nöthnitzer Strasse 40, D-01187 Dresden, Germany
[3]Kristall- und Materiallabor, Physikalisches Institut, Goethe-Universität Frankfurt, Max-von-Laue Stasse 1, 60438 Frankfurt am Main, Germany
[4]Institute for Mathematical Physics, TU Braunschweig, Mendelssohnstraße 3, D-38106 Braunschweig, Germany
[5]Dresden University of Technology, Institute of Solid-State and Material Physics, D-01062 Dresden, Germany
[6]Saint Petersburg State University, Saint Petersburg 198504, Russia
[7]Donostia International Physics Center (DIPC), Departamento de Fisica de Materiales and CFM-MPC UPV/EHU, 20080 San Sebastian, Spain
[8]IKERBASQUE, Basque Foundation for Science, 48011 Bilbao, Spain
[*]*kurt.kummer@esrf.fr*

Supplementary Note 1

Fit analysis of the RXES spectra

For fitting the RXES spectra we used two independent spectral components for the Yb^{2+} and Yb^{3+} emission lines plus a linear background. The line shape of the two spectral components was determined from the experimental data. To this end, we determined the difference between the low and room temperature spectrum of $YbIr_2Si_2$, once normalised to the Yb^{2+} and once to the Yb^{3+} part of the spectrum, as shown in Figure 1. These experimental difference spectra should well reflect pure Yb^{3+} and Yb^{2+} emission, respectively. They are well described by the analytical functions below which we used to fit the experimental data.

The Yb^{2+} line was modelled with the sum of a Lorentzian and a Gaussian line shape

$$f_2(x; E_2) = a_0 \left[(1 - a_1) \exp\left[(- \ln 2 \left(\frac{x - E_2}{a_2} \right)^2 \right] + \frac{a_1}{1 + \left(\frac{x - E_2}{a_2} \right)^2} \right] \quad (1)$$

with $a_0 = 0.2198$, $a_1 = 0.37582$, and $a_2 = 1.859\,\text{eV}$. The asymmetry of the Yb^{3+} line is due to the onset of fluorescence at the absorption edge. It was accounted for by using the sum of a Lorentzian and a Gaussian line shape with a different width and relative weight below and above the white line.

$$f_3(x; E_3) = b_0 \left[(1 - b_1) \exp\left[(- \ln 2 \left(\frac{x - E_3}{b_2} \right)^2 \right] + \frac{b_1}{1 + \left(\frac{x - E_3}{b_2} \right)^2} \right] \quad (2)$$

with $b_0 = 0.1039$ and $b_1 = 0.1390$, $b_2 = 3.5488\,\text{eV}$ for $x \leq x_0$ and $b_1 = 0.5662$, $b_2 = 4.5252\,\text{eV}$ for $x > x_0$. The prefactors a_0 and b_0 ensure that the integral of f_2 and f_3 over all x is one. The small $E2$ component at the low energy side of the RXES spectra, which is due to $2p \to 4f$ quadrupole transitions [1], was modelled by a Gaussian whose position and intensity was fixed with respect to that of the other two components.

$$f_{E2}(x; E_3) = c_1 \exp\left[(- \ln 2 \left(\frac{x - (E_3 - c_2)}{c_3} \right)^2 \right] \quad (3)$$

with $c_1 = 3.93 \times 10^{-3}$, $c_2 = 9.9\,\text{eV}$, and $c_3 = 1.8120\,\text{eV}$.

Using these three components and a linear background we were able to fit the spectra of all measured compounds at all temperatures with no significant differences in the variance of the obtained fit parameters. The fit function applied to the spectra thus has **four free parameters** that describe the intensities (I_2, I_3) and energy position (E_2, E_3) of the Yb^{2+} and Yb^{3+} peaks and two parameters (d, e) for modelling the linear background.

$$f(x; I_2, I_3, E_2, E_3, d, e) = I_2\ f_2(x; E_2) + I_3\ f_3(x; E_3) + (I_3 + I_2)\ f_{E2}(x; E_3) + d\,x + e \quad (4)$$

The least-squares fitting of all spectra using this fit function was numerically very robust and was done by a script with no manual tweaking. Supplementary Figures 2 and 3 show the results of the fit analysis for $YbCo_2Si_2$ and $YbRh_2Si_2$ for all measured temperatures.

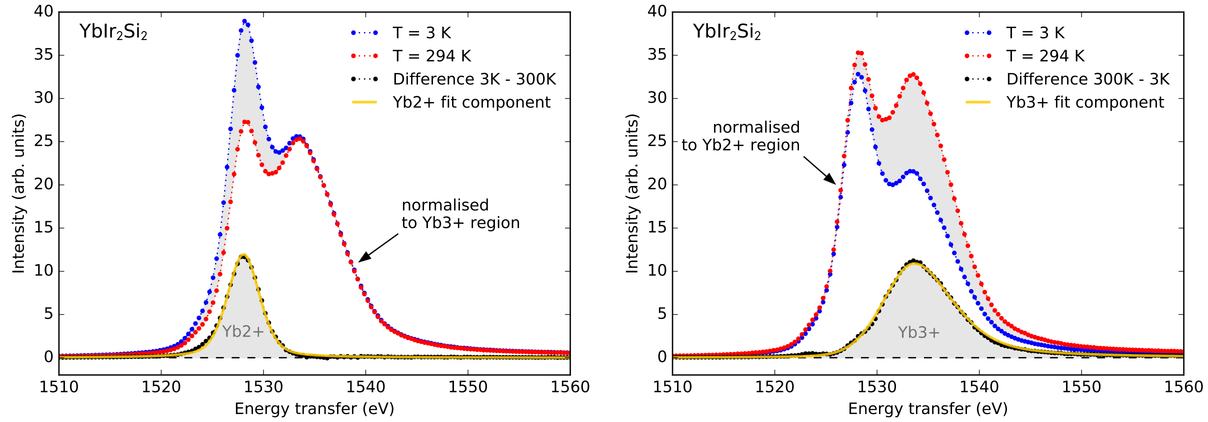

Supplementary Figure 1: Determination of the line shape of the Yb^{2+} and Yb^{3+} spectral components from the difference between the high and low temperature spectra of $YbIr_2Si_2$.

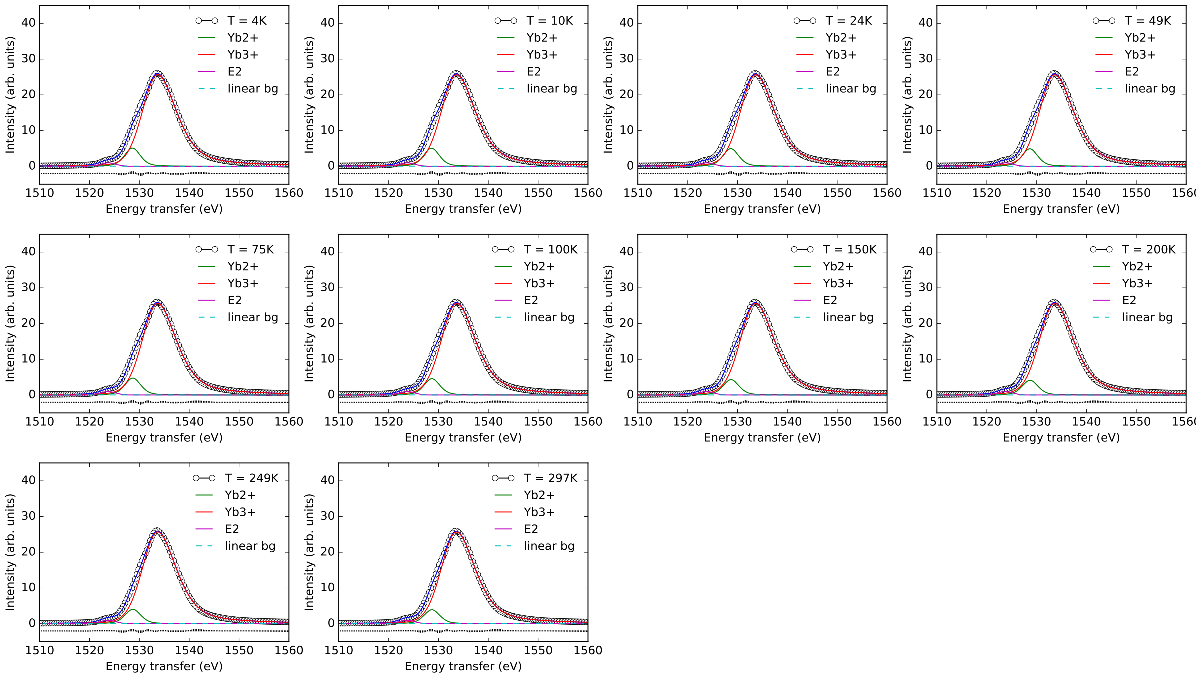

Supplementary Figure 2: Results of the fit analysis of the $YbCo_2Si_2$ RXES spectra for all measured temperatures.

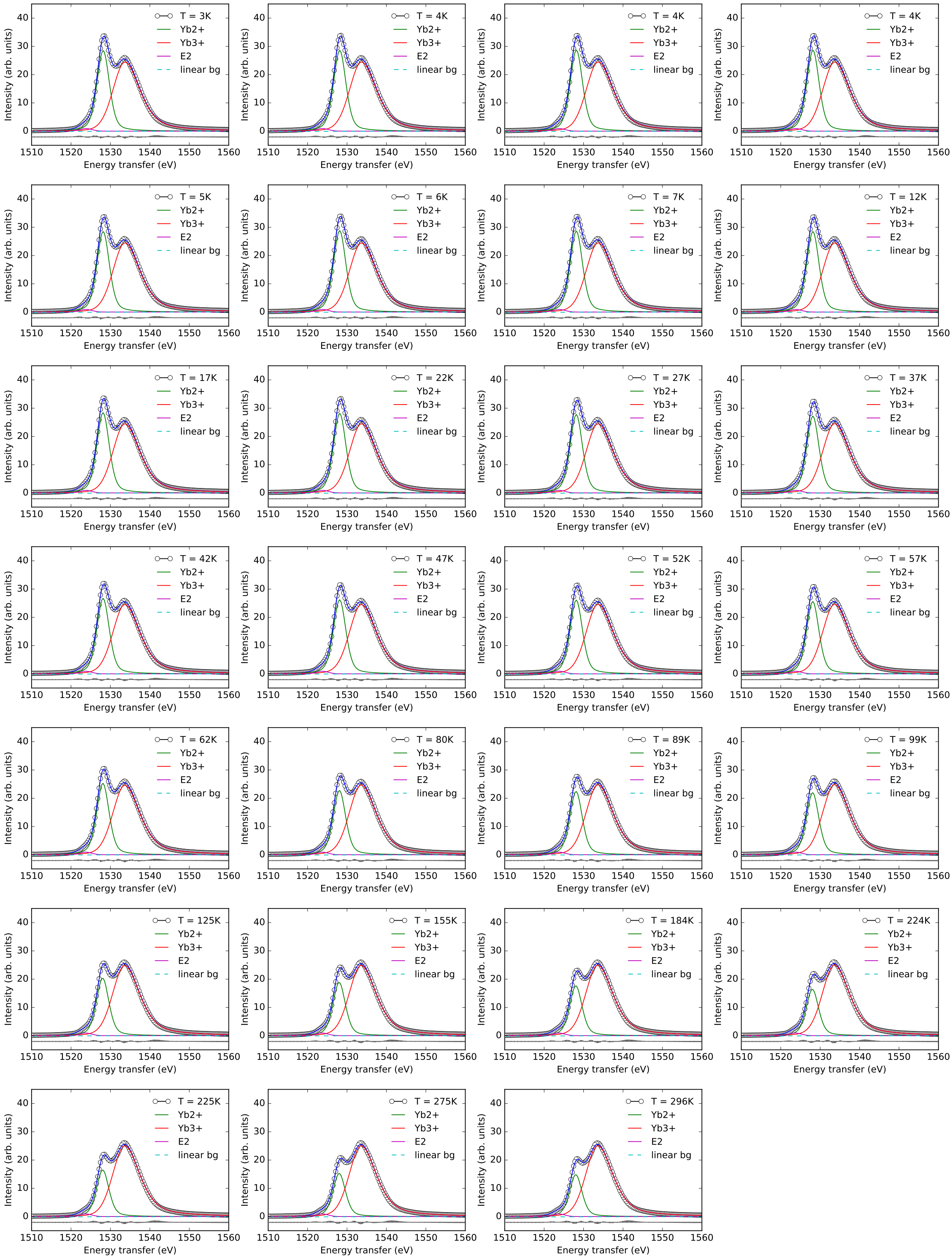

Supplementary Figure 3: Results of the fit analysis of the $YbRh_2Si_2$ RXES spectra for all measured temperatures.

Supplementary Note 2

Determination of the Yb valence from the RXES spectra

From the intensities I_2 and I_3 extracted from the fits of the RXES spectra the Yb valence can be determined as described in [2]. Neglecting the effect of the core hole in the immediate state of the RXES process, $\Delta n_{\mathrm{h}} = 3 - v$ can be estimated as

$$\Delta n_{\mathrm{h}} = \eta = \frac{I_2}{I_2 + \zeta I_3} \qquad (5)$$

The factor $\zeta = 0.13$ accounts for the fact that the Yb^{3+} spectral weight is reduced to 13% in RXES spectra when the incident energy is tuned to the Yb^{2+} resonance (see Figure 7 in [2] and the respective discussion in the text). In the intermediate state with the $2p$ core-hole the two energetically almost degenerate $4f^{14}$ and $4f^{13}$ ground state configurations become energetically well separated with the $2p^5 4f^{14}$ configuration almost 6 eV lower in energy due to the better screening of the core-hole. The spectral weight of the $2+$ component could therefore be larger than the $2+$ weight in the ground state because of the possible $d \to f$ electron hopping. Calculations within the Single Impurity Anderson Model showed that this core hole effect can be taken into account by modifying (5) to

$$\Delta n_{\mathrm{h}} = 0.59\eta + 0.41\eta^2 \qquad (6)$$

In this work, the values reported for Δn_{h} were obtained using (6).

Supplementary Note 3

Relation between hybridization strength, Kondo temperature and valence

The general model to describe the behavior of a system of conduction electrons interacting with localized electrons being at an energy ϵ_f far below the Fermi level is the Anderson model. In this model the strong correlations emerge from the competition between the large on site Coulomb exchange U between $4f$ electrons on the same site and the hybridization between the $4f$ and the conduction electrons, which in the following is parametrized by the bare hybridization strength $\Gamma = \pi\, N(E_{\rm F})\, |V|^2$, with $N(E_{\rm F})$ the conduction electron density of states at the Fermi level and V the hybridization matrix element between f and conduction electrons. The Kondo regime, which is relevant for the systems studied in this work, corresponds to the limit of very large U, i.e. small Γ. In this case the characteristic temperature scale is the Kondo temperature $T_{\rm K}$, which for Yb-systems is typically in the range $0.1\,{\rm K} < T_{\rm K} < 100\,{\rm K}$. In the symmetric and the asymmetric Anderson model it is respectively given by the expressions

$$k_B T_{\rm K} = U\sqrt{\frac{\Gamma}{2U}}\exp\left\{-\frac{\pi U}{8\Gamma}+\frac{\pi\Gamma}{2U}\right\} \quad \text{and} \quad k_B T_{\rm K} = U\sqrt{\frac{\Gamma}{2U}}\exp\left\{-\frac{\pi\,|\epsilon_f|\,|\epsilon_f+U|}{2U\Gamma}\right\} \tag{7}$$

(see [3], Eqs. 6.109 and 6.115). Symmetric and asymmetric cases refer to the position of the Fermi level with respect to the singly occupied and the doubly occupied $4f$ levels.

Since $\Gamma \ll U$, the dependence of $T_{\rm K}$ on Γ is dominated by the exponential term, and therefore $T_{\rm K}$ depends exponentially on the bare hybridization strength Γ. Exact results for physical properties are only available for the case of a single Ce or Yb ion, i.e. ignoring interaction between adjacent rare earth sites, the so-called single impurity case, and ignoring crystal field effects. Then each physical property shows a universal behavior where the only temperature scale is the Kondo temperature. In the Kondo regime, the relation between the valence, i.e. the occupation of the $4f$ level n_f, and the Kondo temperature for $T \to 0$ can be seen rather easily using e.g. the simple approximation to the ground-state wave function suggested by Gunnarsson-Schönhammer [4] for the Anderson impurity with orbital degeneracy N_f, or by the slave-boson mean-field approach for the Kondo lattice (see e.g. [3], Eq. 7.104)

$$n_f(T=0) = \frac{N_f\Gamma/\pi T_{\rm K}}{1+N_f\Gamma/\pi T_{\rm K}} \to 1-\frac{\pi T_{\rm K}}{N_f\Gamma} \tag{8}$$

which implies

$$1-n_f\,(T=0) = \Delta n_f\,(0) \simeq \frac{\pi T_{\rm K}}{N_f\Gamma} \tag{9}$$

Since $T_{\rm K}$ depends exponentially on Γ, i.e. increases much faster than Γ, $\Delta n_f(0)$ should be approximately proportional to $T_{\rm K}$. N. E. Bickers *et al.* numerically calculated $T_{\rm K}$ and n_f for the infinite U Anderson model for several sets of parameter [5]. Plotting their results (table I in [5]) as $\log(\Delta n_f)$ versus $\log(T_{\rm K})$ also results in an almost straight line, with a slope close to what we observed experimentally.

For stronger hybridization, real charge fluctuations becomes relevant leading to an Yb-valence well below 3. This is the mixed-valent regime. There is no strict definition of the border between the Kondo and the mixed-valent regime, since it is a crossover, but commonly this border is set in the range $2.8 > v > 2.7$ (see e.g. [5]). In the mixed-valent regime the valence fluctuations set the dominant energy scale and thus the relevant T scale. It is typically above 300 K, but can also be much higher than 1000 K. Results of numerical calculations for the single impurity case indicate that the characteristic T scale for valence changes with temperature is proportional to the bare hybridization strength Γ, see e.g. Fig. 5.1.a in [7]. Therefore, in the mixed-valent regime the T scale for the variation of the valence with temperature is expected to increase much less with the hybridization strength than in the Kondo regime.

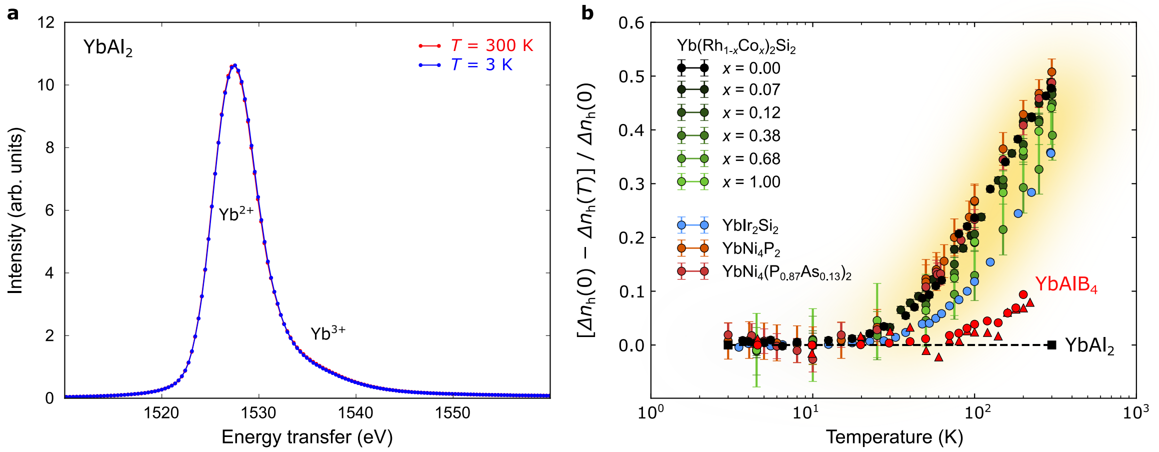

Supplementary Figure 4: (a) Absence of temperature dependent valence changes in the strongly valence-fluctuating $YbAl_2$ up to at least 300 K. (b) Temperature dependence of the valence for the Kondo lattice compounds discussed in the main paper (yellow shaded area) as compared to strongly mixed-valent $YbAl_2$ (see left panel) and $YbAlB_4$ (taken from [6]).

Experimental results show that in strongly mixed-valent Yb systems the change of the valence with temperature is much weaker than in the present systems and shifted to high temperatures. We show here the results for one of the standard strongly mixed-valent system, $YbAl_2$, with $v(T = 0) = 2.2$ [8], as well as for the currently heavily studied system $YbAlB_4$ with $v(T = 0) = 2.75$ [6], which is at the border between the Kondo and the mixed valent regime. The reported temperature dependence for $YbAlB_4$ indeed seems to occur at a higher T scale than that found for the Kondo lattices studied in this work. For $YbAl_2$ no temperature dependence of the valence until at least 300 K has been reported and we observed the same in RXES (see Supplementary Figure 4a).

However, the compounds which are discussed in the main text of this work and which show the universal T behaviour, are all well within the Kondo regime. As we show experimentally, those compounds display a T_v that, surprisingly, is independent of T_K, even when T_K is varied over four orders of magnitude, but at the same time a $\Delta n_\mathrm{h}(T \to 0)$ that scales with T_K as expected.

Supplementary Note 4

Influence of excited CEF levels on the effective Kondo scale

Properties of Ce systems at higher temperatures including the effect of CEF have been theoretically studied using a scaling approach [9, 10, 11]. The result is that at higher temperatures, when the first excited and the second excited CEF doublets get thermally populated, the low temperature Kondo scale $T_{\mathrm{K},0}$, where only the ground state doublet is taken into account, is replaced by an effective high temperature Kondo scale $T_{\mathrm{K},1} = \sqrt{T_{\mathrm{K},0}\,\Delta_1}$ and $T_{\mathrm{K},2} = \sqrt[3]{T_{\mathrm{K},0}\,\Delta_1\,\Delta_2}$, respectively, where Δ_1 and Δ_2 are the energies of the first and the second excited CEF doublets, respectively. Thus, as long as $T_\mathrm{K} \ll \Delta_1 < \Delta_2$, the effective Kondo scale at high T is mostly determined by the CEF splitting, and of the size of the CEF splitting. Further on, the lower $T_{\mathrm{K},0}$, the larger will be the difference between $T_{\mathrm{K},0}$ and $T_{\mathrm{K},2}$. For trivalent Yb the effect is even stronger, because the ground state J multiplet is $J = 7/2$ instead of $J = 5/2$ for Ce. Thus the CEF leads to four doublets instead of three. Accordingly the high temperature Kondo scale will be $T_{\mathrm{K},3} = \sqrt[4]{T_{\mathrm{K},0}\,\Delta_1\,\Delta_2\,\Delta_3}$.

Usually the Kondo effect is considered to become less and less effective upon increasing T, but there are some properties for which a strengthening of the effect of the Kondo interaction upon increasing T due to the involvement of excited CEF levels is well known and theoretically and experimentally well documented, e.g. the resistivity [12, 13]. For the simple, but theoretically more accessible single ion Kondo model, the magnetic contribution to the resistivity $\rho_m(T)$ first decreases logarithmically upon increasing T, with parameters defined by $T_{\mathrm{K},0}$. But when T approaches Δ_1 and/or Δ_2, $\rho(T)$ increases markedly, passes through a maximum, and then decreases again logarithmically, but with a larger slope than at lower T. The larger slope at high T is directly connected to the higher effective degeneracy of the local moment at high T [12]. A similar behavior is also expected for the Kondo lattice [13]. Furthermore, the high temperature Kondo scale defined above, $T_{\mathrm{K},1}$ and $T_{\mathrm{K},2}$, have been shown to be relevant for the description of the magnetic susceptibility at high T [11].

Supplementary Note 5

Kondo lattice

We note that the theoretical calculations we refer to for the evolution of $n_f(T)$ with T_K deal with the single ion Kondo model, not the dense Kondo lattice. The latter has the additional restriction that the total number of electrons has to be constant, and therefore the position of the f level has to be shifted with T. Unfortunately a calculation of $n_f(T)$ for a Kondo lattice and including the CEF seems to be beyond present theoretical possibilities. However, differences between the single ion Kondo system and the Kondo lattice are usually associated with the onset of coherence at low T, and are therefore unlikely to significantly affect the high T regime. Furthermore the temperature T_coh at which coherence sets in is related to the Kondo scale and differs by as much as T_K in the systems studied here. Therefore it is unlikely that the formation of a coherent state is at the origin of the nearly identical T_v in systems with such different T_coh.

Supplementary Note 6

Extraction of T_{v} from the experimental data

In the manuscript, we fitted a previously used analytic expression to the curves Δn_{h} vs. T in Fig. 3 in order to assign a characteristic temperature scale T_{v} to each curve. The T_{v} extracted in this way satisfy $\Delta n_{\mathrm{h}}(T_{\mathrm{v}}) = 1/2\,\Delta n_{\mathrm{h}}(T \to 0)$ which is a common way of defining T_{v}, and allows direct comparison with the T_{v} scale reported in other experimental and theoretical studies (see for instance Fig. 14 in [5]). The advantage of the used analytical expression is that it only depends on $\Delta n_{\mathrm{h}}(0)$ and T_{v}, the two parameters of interest, with no additional free parameters. However, other ways of extracting T_{v} could be chosen. Here we show that our findings are not affected by the way in which T_{v} is extracted from the experimental data.

As an example we show in Figure 5(a) straight line fits to the high temperature part of the $\Delta n_{\mathrm{h}}(T)$. We then identify T_{v} with the value at which the straight line cuts $\Delta n_{\mathrm{h}}(0)$, corresponding to the onset of valence changes with T, or $1/2\,\Delta n_{\mathrm{h}}(0)$ corresponding to the usual assignment of T_{v} , respectively. $\Delta n_{\mathrm{h}}(0)$ is in both cases obtained from a constant line fit to the low temperature part of the curves which means three free fitting parameters in total (slope and intercept for the straight line and intercept for the constant). The T_{v} obtained in this way are shown in Figure 5b, overlaid with the numbers reported in the main manuscript. The numbers obtained using the intercept with the $1/2\,\Delta n_{\mathrm{h}}(0)$ line are the same as those reported in the main manuscript. Naturally, the intercept of the straight line fits with $\Delta n_{\mathrm{h}}(0)$, marking the onset of temperature dependence, yields lower absolute values for T_{v} in comparison, approximately one order of magnitude lower than those reported in the manuscript. However, also with this definition the T_{v} values of all analyzed compounds are very similar and do not differ by more than a factor of 2, despite largely different T_{K}'s. Thus the main conclusion of our paper does not depend on how T_{v} is defined.

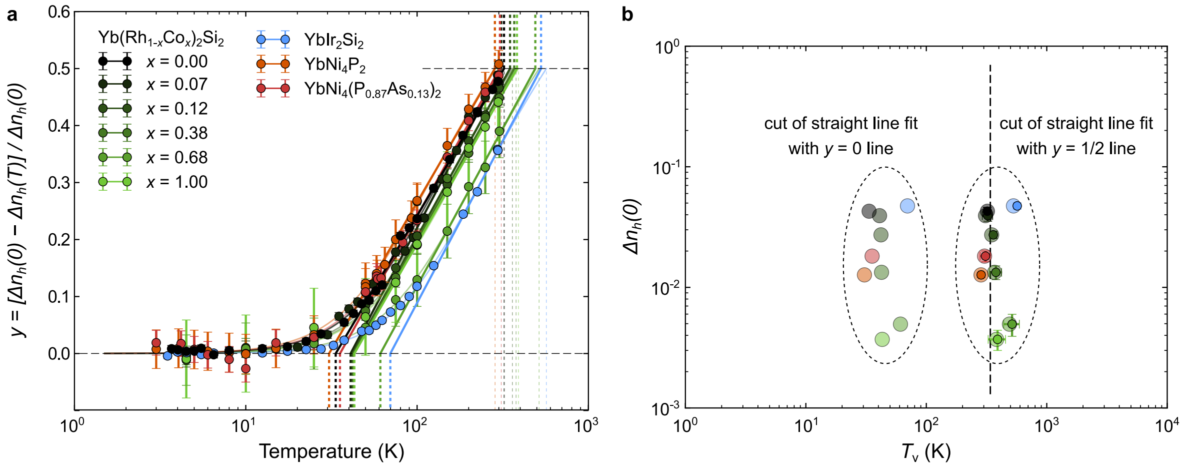

Supplementary Figure 5: (a) Straight line fits to the high temperature part of the experimental curves. (b) T_{v} values from the straight line fits using once the temperature at which y cuts zero and $y = 1/2$, respectively (semitransparent circles). The values found for $y = 1/2$ agree with those reported in the paper (opaque circles) within the errorbars. Using the onset of temperature changes, i.e. the cut of the straight line with $y = 0$, yields for all compounds T_{v} values of about 30 K for all compounds. The observation of a universal temperature scale independent of T_{K} does not depend on how exactly T_{v} is defined or extracted.

Supplementary References

[1] Dallera, C. *et al.* New spectroscopy solves an old puzzle: The Kondo scale in heavy fermions. *Phys. Rev. Lett.* **88**, 196403 (2002).

[2] Kummer, K. *et al.* Intermediate valence in Yb compounds probed by $4f$ photoemission and resonant inelastic x-ray scattering. *Phys. Rev. B* **84**, 245114 (2011).

[3] Hewson, A. C. *The Kondo problem to heavy fermions* (Cambridge University Press, 1997).

[4] Gunnarsson, O. & Schönhammer, K. Electron spectroscopies for Ce compounds in the impurity model. *Phys. Rev. B* **28**, 4315–4341 (1983).

[5] Bickers, N. E., Cox, D. L. & Wilkins, J. W. Self-consistent large-n expansion for normal-state properties of dilute magnetic alloys. *Phys. Rev. B* **36**, 2036–2079 (1987).

[6] Matsuda, Y. H. *et al.* Synchrotron x-ray spectroscopy study on the valence state in α- and β-$YbAlB_4$ at low temperatures and high magnetic fields. *J. Korean Phys. Soc.* **62**, 1778–1781 (2013).

[7] Schlottmann, P. Some exact results for dilute mixed-valent and heavy-fermion systems. *Physics Reports* **181**, 1 – 119 (1989).

[8] Matsunami, M. *et al.* Photoemission evidence for valence fluctuations and Kondo resonance in $YbAl_2$. *J. Phys. Soc. Jpn.* **81**, 073702 (2012). https://doi.org/10.1143/JPSJ.81.073702.

[9] Yamada, K., Yosida, K. & Hanzawa, K. Comments on the dense Kondo state. *Prog. Theor. Phys.* **71**, 450–457 (1984).

[10] Hanzawa, K., Yamada, K. & Yosida, K. Orbital degeneracy effect on the dense Kondo state in real systems. *J. Magn. Magn. Mater.* **47**, 357 – 359 (1985).

[11] Aviani, I. *et al.* Kondo effect in $Ce_xLa_{1-x}Cu_{2.05}Si_2$ intermetallics. *Phys. Rev. B* **64**, 184438 (2001).

[12] Cornut, B. & Coqblin, B. Influence of the crystalline field on the Kondo effect of alloys and compounds with cerium impurities. *Phys. Rev. B* **5**, 4541–4561 (1972).

[13] Lassailly, Y., Bhattacharjee, A. K. & Coqblin, B. Low-temperature resistivity and magnetoresistivity of cerium compounds. *Phys. Rev. B* **31**, 7424–7429 (1985).
